# Urine tenofovir testing for real‐time PrEP adherence feedback: a qualitative study involving transgender women in Uganda

**DOI:** 10.1002/jia2.26255

**Published:** 2024-05-02

**Authors:** Andrew Mujugira, Beyonce Karungi, Jackson Mugisha, Agnes Nakyanzi, Olivia Nampewo, Faith Naddunga, Brenda Kamusiime, Rogers Nsubuga, Kikulwe R. Nyanzi, Timothy R. Muwonge, Monique A. Wyatt, Norma C. Ware, Monica Gandhi, Jessica E. Haberer

**Affiliations:** ^1^ The Infectious Diseases Institute Limited Makerere University Kampala Uganda; ^2^ Department of Global Health University of Washington Seattle Washington USA; ^3^ Transgender Equality Uganda Kampala Uganda; ^4^ Department of Global Health and Social Medicine Harvard Medical School Boston Massachusetts USA; ^5^ Harvard Global Cambridge Massachusetts USA; ^6^ Department of Medicine Brigham and Women's Hospital Boston Massachusetts USA; ^7^ Department of Medicine University of California San Francisco San Francisco California USA; ^8^ Harvard Medical School Boston Massachusetts USA; ^9^ Centre for Global Health Massachusetts General Hospital Boston Massachusetts USA

**Keywords:** urine tenofovir test, pre‐exposure prophylaxis, adherence, transgender women, qualitative, Africa

## Abstract

**Introduction:**

Adherence counselling with point‐of‐care (POC) drug‐level feedback using a novel tenofovir assay may support pre‐exposure prophylaxis (PrEP) adherence; however, perceptions of urine testing and its impact on adherence are not well studied. We qualitatively examined how POC tenofovir testing was experienced by transgender women (TGW) in Uganda.

**Methods:**

Within a cluster randomized trial of peer‐delivered HIV self‐testing, self‐sampling for sexually transmitted infections and PrEP among HIV‐negative TGW showing overall low PrEP prevention‐effective adherence (NCT04328025), we conducted a nested qualitative sub‐study of the urine POC assay among a random sample of 30 TGW (August 2021−February 2022). TGW interviews explored: (1) experiences with POC urine tenofovir testing and (2) perceptions of PrEP adherence counselling with drug‐level feedback. We used an inductive content analytic approach for analysis.

**Results:**

Median age was 21 years (interquartile range 20–24), and 70% engaged in sex work. Four content categories describe how TGW experienced POC urine tenofovir testing: (1) Urine tenofovir testing was initially met with scepticism: Testing urine to detect PrEP initially induced anxiety, with some perceptions of being intrusive and unwarranted. With counselling, however, participants found POC testing acceptable and beneficial. (2) Alignment of urine test results and adherence behaviours: Drug‐level feedback aligned with what TGW knew about their adherence. Concurrence between pill taking and tenofovir detection in urine reinforced confidence in test accuracy. (3) Interpretation of urine tenofovir results: TGW familiar with the interpretation of oral‐fluid HIV self‐tests knew that two lines on the test device signified positivity (presence of HIV). However, two lines on the urine test strip indicated a positive result for non‐adherence (absence of tenofovir), causing confusion. Research nurses explained the difference in test interpretation to participants’ satisfaction. (4) White coat dosing: Some TGW deliberately chose not to attend scheduled clinic appointments to avoid detecting their PrEP non‐adherence during urine testing. They restarted PrEP before returning to clinic, a behaviour called “white coat dosing.”

**Conclusions:**

Incorporating POC urine testing into routine PrEP adherence counselling was acceptable and potentially beneficial for TGW but required attention to context. Additional research is needed to identify effective strategies for optimizing adherence monitoring and counselling for this population.

## 1 INTRODUCTION

Transgender women (TGW), who are biologically assigned male at birth but identify as female, face a significant burden of HIV in East and Southern Africa. The reported prevalence of HIV among TGW in this region varies, ranging from 9.4% in Rwanda to 63.5% in South Africa [1]. In sub‐Saharan Africa, the standardized prevalence of HIV among TGW is estimated to be 29.9%, and TGW aged 15 or older have an overall odds ratio for HIV acquisition of 21.5 [2]. The elevated prevalence of HIV among TGW can be attributed to multi‐level barriers [3]. Structural issues such as discriminatory practices, limited employment opportunities, poor access to healthcare, legal barriers and mental health challenges contribute to increased engagement in high‐risk behaviours such as condomless receptive anal intercourse, transactional sex and sharing needles for hormone injections [4, 5, 6]. Client‐centred interventions can effectively provide comprehensive support for TGW to reduce their vulnerability to HIV acquisition and promote overall wellbeing.

Oral pre‐exposure prophylaxis (PrEP) is an efficacious method for preventing HIV transmission; however, its effectiveness depends on adequate adherence [7]. TGW face unique multi‐level intersecting barriers that hinder their engagement in PrEP care, including concerns about potential interactions between PrEP and gender‐affirming hormones, lack of social support, substance use, mental health concerns, incarceration, transphobia, healthcare stigma and avoidance and HIV‐related stigma [3, 8, 9, 10]. Data from Zimbabwe, for instance, indicate that only 60% of HIV‐negative TGW were aware of PrEP in 2019, of whom 39% had ever used it [11]. These gaps in the PrEP cascade highlight the need for tailored implementation strategies to facilitate PrEP utilization among TGW [12].

A recently developed point‐of‐care (POC) immunoassay for quantifying tenofovir levels in urine has enabled real‐time monitoring of PrEP adherence within the previous 48−72 hours [13]. Detection of non‐adherence through urine testing, followed by adherence counselling, is associated with improved PrEP adherence [14, 15, 16]. However, perceptions of the testing and its impact on adherence have not been studied among TGW [17, 18, 19]. To address this gap, we conducted a qualitative study within a randomized trial testing the effect of peer‐delivered HIV self‐testing (HIVST) and sexually transmitted infection self‐sampling (STISS) on oral PrEP adherence in Uganda. The primary results of the trial revealed low PrEP prevention‐effective adherence; ≤10% had tenofovir diphosphate levels ≥700 fmol/punch at any visit [20]. The present analysis explored TGW experiences with POC urine tenofovir testing and perceived influences of real‐time drug‐level feedback on adherence behaviour.

## 2 METHODS

### 2.1 Population and procedures

The Peer Study was a pilot cluster randomized trial designed to assess the effectiveness of HIVST and STISS, delivered by peers, in promoting PrEP uptake among TGW in Kampala, Uganda. The study was conducted between October 2020 and July 2022 and is registered on ClinicalTrials.gov under the identifier NCT04328025. Before the trial was implemented, 20 qualitative interviews with TGW peers were conducted to identify preferences for peer‐delivered combination prevention [21]. The formative research identified peer confidentiality, trustworthiness and practical knowledge of biomedical HIV prevention tools as facilitators of HIV/Sexually Transmitted Infections (STI) testing and PrEP use. The cluster Randomized Controlled Trial (RCT) consisted of 10 TGW peer groups, each with one peer and eight TGW. Peer groups were randomly assigned in a 1:1 ratio to receive either monthly delivery of HIVST, STISS and PrEP by peers (intervention arm) or quarterly in‐clinic HIV/STI testing and PrEP prescription (standard of care [SOC]). This project followed a community‐based participatory research approach [22], which involved collaborating with TGW civil society organizations, consulting with TGW during the planning and protocol development phases, and selecting and training TGW peers to lead recruitment and retention efforts. Furthermore, this approach included TGW representation on the research team and community advisory group. To ensure equitable involvement throughout the trial, potential power dynamics were addressed through open discussions with TGW participants and peers.

TGW participants were recruited through peer‐led snowball sampling and enrolled at the research clinic, where they were initiated on PrEP. They attended scheduled visits at the clinic every 3 months and were followed for 12 months. At each visit, they underwent HIV and STI testing, with free treatment provided for *Neisseria gonorrhoeae* (NG) and *Chlamydia trachomatis* (CT), along with individualized counselling on HIV risk reduction. In addition, participants received free condoms and lubricant as SOC. They were also given four OraQuick® HIV self‐test kits (OraSure Technologies, USA), which they could use or share with their committed intimate partners. At quarterly visits, all participants received three bottles of PrEP medication, each containing a 1‐month supply of lamivudine/tenofovir disoproxil fumarate.

### 2.2 Intervention delivery

Trial procedures are reported elsewhere [23]. Briefly, TGW peers were identified during formative research and subsequently received a 2‐week training by study staff to deliver the intervention. The training curriculum was developed based on findings from the formative phase. It encompassed areas such as HIV self‐test usage and result interpretation, self‐collection of oro‐pharyngeal, rectal and urine specimens using visual guides for self‐collected swabs, distribution of HIVST and STISS kits, refills of PrEP medication and linkage of TGW who tested positive into HIV care. At enrolment, participants were introduced to their designated TGW peer, with mutual sharing of phone numbers upon consent. To facilitate communication between peers and participants, the study provided peers with dedicated phones equipped with pre‐paid voice/data bundles that were replenished monthly. Additionally, trained peers regularly contacted TGW in their assigned group between quarterly visits to schedule individual meetings at a location convenient for the participant (e.g. home, shelter, workplace).

The peer intervention included the following components: (a) providing additional HIV self‐test kits and PrEP refills; (b) instructing TGW on how to use STI self‐sampling kits to collect oro‐pharyngeal, rectal and urinary specimens, which were then delivered to the research clinic on the same day; and (c) offering psychosocial support for adherence to PrEP, HIVST with intimate partners and peer counselling according to national guidelines [24]. All participants received free testing and treatment for CT and NG. Quarterly assessments of HIVST, STISS, PrEP adherence and sexual risk behaviours were conducted using interviewer‐administered questionnaires. Furthermore, all participants received individualized counselling on reducing their risk of acquiring HIV and free condoms and lubricants as SOC. The design of this intervention was informed by the Social Ecological Framework for PrEP introduction [25]. The trial's primary outcome was PrEP adherence measured through urine tenofovir detection and tenofovir diphosphate levels in dried blood spot samples collected quarterly and batch‐tested [26]. Overall, PrEP adherence was low—≤15% had detectable tenofovir‐diphosphate levels at any visit—with no difference by randomization arm (*p* = 0.18) [27].

### 2.3 Adherence counselling

Study nurses utilized the Integrated Next Step Counseling (iNSC) approach, a strength‐based, non‐judgemental discussion to identify the sexual health and PrEP adherence needs of TGW and tailoring strategies to meet these needs [28]. Counselling sessions aimed to: (1) support TGW in adhering to PrEP if desired and (2) promote sexual health as an integral part of self‐care and overall wellbeing. This counselling included assessing the participant's comprehension of PrEP, identifying barriers to adherence, discussing potential side effects, addressing perceived obstacles to adherence and evaluating self‐reported adherence. Nurses were trained and mentored by an experienced iNSC trainer, who conducted on‐site assessments to witness their implementation of iNSC and provided real‐time feedback on their performance. Because iNSC was developed in the United States, adaptations to iNSC were made for the Ugandan context through discussion among Ugandan investigators, counsellors and TGW community members. These adaptations retained the model's underpinnings while accounting for cultural norms and expectations that are relatively more directive around shared problem‐solving tailored to individual needs versus explicit shared decision‐making.

### 2.4 Urine tenofovir testing

TGW attended quarterly clinic appointments, at which they were reminded that a urine sample would be collected to measure tenofovir levels, as stated in the informed consent form. During the appointment, the nurse requested that TGW provide a urine sample, which was tested in their presence. Since the test uses a competitive assay in its prototype form, the nurse explained that two lines on the test strip indicated a positive test for non‐adherence (i.e. absence of tenofovir). In contrast, one line signified a negative test result (drug detection). Based on this drug‐level feedback, individualized PrEP adherence counselling was tailored based on the participants’ test results, including positive reinforcement to foster adherence and a “de‐shaming” communication approach when non‐adherence was identified [29].

### 2.5 Data collection

Qualitative findings about participation in the trial based on in‐depth interviews with TGW, intimate partners and TGW peers are published separately [23]. We randomly selected 30 TGW from the 41 participants in the peer intervention group. Three TGW could not be contacted for interviews and were substituted with other trial participants through snowball sampling. Each TGW in the nested study participated in one qualitative interview, which lasted about 60 minutes and explored experiences of urine tenofovir testing and real‐time drug‐level feedback. All 10 peers involved in intervention delivery were also interviewed in the parent study. Interviews were conducted by qualified social scientists (AN, BK, CCT, GKN, JM and VK) in English or Luganda (the local language) at locations chosen by the participants to ensure privacy and confidentiality. Interviews were audio‐recorded with the participant's permission and transcribed into English by the interviewer, who also wrote field notes. We conducted thorough quality checks on all transcripts, correcting and revising any errors found; transcripts were not returned to TGW. Every participant received a reimbursement of UGX 30,000 (equivalent to US$8.10) approved by the local Institutional Review Board.

### 2.6 Data analysis

The present analysis focuses on urine tenofovir testing and adherence counselling. We used an inductive, content analytic approach to data analysis as previously described [30]. Analysis began with repeated reviews of interview transcripts to gain familiarity with the interview content. Following the initial assessment, open coding involved a team of six coders (AM, AN, BK, CCT, JM and VK) who iteratively identified and labelled relevant content to determine specific text sections that shed light on experiences of urine tenofovir testing drug‐level feedback, and adherence behaviours related to testing. The coders utilized a codebook consisting of provisional labels that were defined and illustrated to guide their coding. The data were organized using Dedoose software (SCRC, Hermosa Beach, CA). Once complete, the coded data were sorted into categories based on the initial concepts identified by the coders. These categories were developed inductively and encompassed all primary themes present in the data. The COREQ checklist was used to ensure accurate reporting of the study findings [31].

### 2.7 Ethics approval

The Mildmay Uganda Research Ethics Committee (0304‐2019), Partners Human Research Committee (Massachusetts General Hospital; 2019P001620) and Uganda National Council for Science and Technology (HS390ES) all approved this study. Written informed consent was obtained from each qualitative study participant in English or Luganda.

## 3 RESULTS

### 3.1 Participant characteristics

The median age of the 30 TGW was 21 years (interquartile range [IQR] 20−24). They reported a median of 11 years of schooling (IQR 9−13). The median monthly income was UGX 200,000 ($53.99) (Table 1); Uganda's average monthly income at the time of the study was UGX 416,000 ($107.74) [32]. The median number of anal sex acts in the previous month was 10 (IQR 5−15). Twenty‐seven participants (90%) had access to lubricants, 25 (83%) reported having access to condoms and 20 (70%) engaged in sex work. Among those reporting engagement in sex work, the median charge for anal sex was UGX 50,000 ($13.50) when a condom was used and UGX 62,500 ($16.90) without a condom. Additionally, 20 TGW (67%) disclosed that they were regularly using alcohol.

**Table 1:** Baseline characteristics of TGW participants (*N* = 30 unless otherwise indicated)

Characteristic	Median (IQR) or *N* (%)
Age (years)	
<25	25 (83)
≥25	5 (17)
Education (years)	
≤10	11 (37)
>10	19 (63)
Monthly income (UGX)	200,000 (100,000, 300,000)
Partnership status	
Intimate partner	20 (67)
No intimate partner	10 (33)
Anal sex acts, prior month (*n* = 21)^a^	10 (5,15)
Access to condoms	25 (83)
Access to lubricants	27 (90)
Sells sex for money or goods	21 (70)
Charge for anal sex with a condom (*n* = 11)^a^	UGX 50,000 (30,000, 100,000)
Charge for anal sex without a condom (*n* = 10)^a^	UGX 62,500 (40,000, 200,000)
Currently taking alcohol	20 (67%)
Currently taking tobacco	2 (7%)
Currently using recreational drugs	1 (3%)
Ever used hormone replacement therapy (*n* = 16)^a^	0 (0%)
Ever incarcerated^a^	7 (23%)
Ever incarcerated for being transgender (*n* = 7)	3 (43%)

*Note*: Quantitative data derived from parent clinical trial.

Abbreviations: IQR, interquartile range; UGX, Uganda Shillings.

^a^ Some participants declined to answer questions.

### 3.2 Qualitative results

The qualitative analysis yielded four distinct content categories. In the first category (1. “Urine tenofovir testing was met with scepticism…”), we see that participating TGW initially experienced test anxiety when undergoing urine tenofovir testing. However, with the support of counselling, they came to perceive testing as acceptable and beneficial. The second category (2. “Alignment between drug‐level feedback…”) shows how receiving feedback from the urine test that was consistent with pill‐taking behaviours increased trust in the accuracy of the results. In category 3 (3. “Interpretation of urine tenofovir results…”), we see how initial confusion among participants regarding differences in interpretation between POC HIV tests and urine tenofovir tests was effectively addressed by research nurses. The final category (4. “White coat dosing…”) shows how some transwomen changed their dosing habits to appear adherent by taking PrEP shortly before their scheduled clinic visits to avoid any potentially unfavourable test results.

1. Urine tenofovir testing was met with scepticism but eventually gained acceptance.

TGW were unaccustomed to having their tenofovir levels evaluated through urine testing, making this experience new and stressful, as previous studies had primarily utilized batch testing of blood for measuring tenofovir levels. Initially, some clients perceived urine testing as intrusive—an indicator of lack of trust in their self‐reported adherence (“*Why do you want to test my urine to see if I take PrEP or not?*” TGW, age 20). Furthermore, participants were concerned about how the test worked, especially given its novelty. They believed their confusion prompted research nurses to explain that after being ingested, tenofovir was absorbed into the bloodstream and subsequently excreted through the urine, enabling its detection in both body fluids.

*“When she asked me to provide my urine sample for testing, I was surprised. I didn't trust it because I thought PrEP was found in the blood. But how come it is tested in the urine? ‘How is it possible? How does this thing work?’ Then she explained that urine can be used to test for PrEP, which is why we have used it.”* (TGW, age 22)

*“I was a bit scared…How is it possible for her to detect PrEP in my urine? Yet I take the pills. At first, I doubted it. Then I thought, it might be [a] new innovation or technology.”* (TGW, age 22)

Participants felt less anxious when nurses offered counselling to alleviate uncertainty and concerns about the new urine tenofovir test. They felt reassured when nurses explained that the primary purpose of the testing was to ensure the participant was taking PrEP to protect them from HIV, which helped to dissipate feelings of anger when asked for a urine sample. TGW regarded POC testing as beneficial for their health and wellbeing: *“I was happy when they told me that there is a testing kit to detect PrEP in someone's body” (TGW, age 19)*. Counselling also fostered a sense of care and concern, leading TGW to regard this testing approach as acceptable.

*“She called me and explained everything to me. I quarrelled with her. ‘This is my life. Why are you so concerned?’ Then she asked me to calm down. She told me, ‘We don't want you to pretend you are taking PrEP when you are not.’ I exchanged words with her, but she calmed me down and explained that ‘If you are not taking PrEP, you are not safe, and we can counsel you if you find it hard to take PrEP.’ Then I accepted and gave her my urine sample for testing.”* (TGW, age 20)

*“It is not for their own good. It is for my good, so I willingly gave my urine to test if I take PrEP. And if I am not taking PrEP, the doctor can be my witness. She advised me that stopping PrEP is not a good thing. So, I continued taking my pills.”* (TGW, age 19).

2. Alignment between drug‐level feedback and pill‐taking behaviours strengthened trust in urine tenofovir testing.

TGW participants identified the urine tenofovir test as accurate when drug‐level feedback aligned with the knowledge of their pill adherence. This confirmation bolstered their confidence in the test's reliability. Furthermore, observing their test results led TGW individuals to believe in the effectiveness of the test and increased their trust in real‐time monitoring of their adherence. None described discrepancies between self‐reported adherence and urine test results.

*“…when we were arrested…I wasn't taking my pills. So, when I came back for the clinic visit, the health worker asked me to provide a urine sample. When she showed me the results, there was no PrEP. I could also see it by myself. I trust it, and it works because when they tested my urine, it is true I was not taking my pills, and the results showed no PrEP in it.”* (TGW, age 21)

*“I think urine testing is authentic because the first time I did it, I was not on PrEP, but it came out negative. The PrEP was not present. The second time I went there, I was taking PrEP, and she told me I was taking PrEP. So, I think urine tests are authentic and work.”* (TGW, age 25)

The provision of drug‐level feedback served as a motivator for enhancing PrEP adherence. Among TGW individuals who self‐identified as adherent to PrEP and disclosed potential exposure to HIV, the confirmation of tenofovir detection through their urine POC test strengthened their commitment to continuing PrEP use. Additionally, the combination of a negative HIV self‐test result and the presence of tenofovir in urine further reinforced their confidence in the effectiveness of PrEP as a preventive measure against HIV acquisition.

*“We sex workers meet 2–3 persons a day but don't know their HIV status. I have not been testing them. I have sex with them and leave. I think PrEP has been helping me because one day, musawo [nurse] tested me to see if I take PrEP. Results showed that I was taking it very well, and she requested me to continue taking it well. It prevents one from getting HIV.”* (TGW, age 22)

*“When I test myself for HIV, I don't have it. When they tested for PrEP, it showed that it was there. [The nurse] thanked me for taking my pills very well because PrEP was detected in my urine. Then I felt happy, and I was like, ‘Oh, musawo has appreciated that I do take PrEP.’”* (TGW, age 22)

3. Interpretation of urine tenofovir results differed from HIV self‐tests, initially causing confusion.

TGW familiar with interpreting oral‐fluid HIV self‐tests were aware that the test device displayed two lines to indicate a positive test result and the presence of HIV. In comparison, the urine tenofovir test (which currently uses competitive assay technology) showed two lines for a positive result of non‐adherence, which indicated the absence of tenofovir. This scenario confused TGW, who had been exposed to both POC testing strategies and intuitively understood that a positive result indicated the presence of something. Participants reported gaining comprehension and satisfaction when research nurses thoroughly explained the differences in test interpretation.

*“It really confused me because, with the HIV self‐test kit, the signs are not the same as this one. Then she explained it to me. I cannot confuse the two because she explained it to me very well, and I understood it.”* (TGW, age 20)

*“I thought it is a kit for testing HIV. It confused me…but it showed that I don't take PrEP.”* (TGW, age 21)

4. White coat dosing was used to conceal PrEP non‐adherence.

Some TGW deliberately missed scheduled clinic visits to conceal their non‐adherence to PrEP. This behaviour stemmed from the belief that maintaining the appearance of adherence was crucial for remaining in the study. These individuals had misconceptions about the purpose of urine testing, mistakenly thinking that a negative result would result in being removed from the study and losing potential benefits from research participation. These participants were known by fellow TGW peers to strategically resume PrEP usage shortly before returning to the clinic, a practice commonly referred to as “white coat dosing.”

*“I called her and said go to [the clinic]. She told me that whenever she comes for clinic visits, they first test if she takes PrEP. She told me that she would not come because she had not been taking PrEP; I should wait for two weeks. By then, she would have taken PrEP, and it is detected.”* (Peer 03, age 22)

*“There is a friend of mine whom I advised to take her pills daily because she could miss taking them. One day she came back here for her clinic visits, and she was so worried when she was told that they were going to test for tenofovir in her urine. She thought that they were going to discontinue her from the study because she was not taking PrEP. Good enough, she followed my advice, and right now, she takes her pills very well.”* (TGW, age 22).

Study nurses provided adherence counselling tailored to the participant's needs. This included emphasizing that PrEP use was not a lifelong commitment, but rather limited to periods of risk. For instance, participants could discontinue PrEP when not engaging in sexual activity. TGW were aware that taking PrEP and urine tenofovir testing was voluntary. As a result, some individuals chose not to participate in urine tenofovir testing when requested for samples.

*“…at the beginning, I took [PrEP], then I stopped. I had a partner, then we broke up, and I was like, ‘I think I am done with sex for some time’. I told [the nurse] that I am not taking PrEP because I am not sexually active. She said, ‘When you feel like you want to take it again, you can come’.”* (TGW, age 25)

*“I remember musawo [nurse] told me that ‘there are some transwomen we ask to provide their urine sample and they refuse.’ But I was not scared [of testing] because I knew I was taking my PrEP.”* (TGW, age 19)

## 4 DISCUSSION

This qualitative research conducted among TGW participating in a cluster randomized trial in Kampala, Uganda, found that POC urine tenofovir testing and real‐time drug‐level feedback initially caused anxiety. However, upon receiving counselling and drug‐level feedback that confirmed their perspectives on pill‐taking and reassurance about the purpose of urine testing, participants became more confident in its accuracy. Participants familiar with interpreting oral‐fluid HIV self‐tests understood that two lines on the test device indicated the presence of HIV. However, there was confusion when the visual display of two lines on the urine tenofovir test strip signified the absence of tenofovir. Some TGW purposely skipped clinic visits when they were aware that their urine test would reveal non‐adherence to PrEP, only to resume PrEP usage before returning to the clinic to avoid a negative test result. Overall, with explanations and supportive counselling from study staff, POC urine testing was perceived as novel, acceptable and accurate, motivating PrEP use among TGW with HIV exposure.

To our knowledge, no published qualitative studies have evaluated how urine tenofovir testing and drug‐level feedback to support PrEP adherence are experienced by TGW in any setting. However, a cross‐sectional survey conducted in the United States with 40 PrEP users (including three who identified as transgender) revealed that urine tenofovir testing was highly acceptable [33]. Other studies conducted among those on antiretroviral treatment in South Africa [19] and female PrEP users in Kenya [18] confirmed the acceptability of the urine test. Our research findings align with these studies and a similar one involving 100 TGW and men prescribed PrEP in the United States, which reported a strong correlation between self‐reported adherence to PrEP and the detection of tenofovir in urine samples [34]. Our finding of initially negative emotions and distrust in POC urine testing underscores the importance of tailored adherence counselling. This approach helped TGW to perceive the urine POC test as acceptable and beneficial. Future studies should evaluate strategies to optimize PrEP adherence monitoring and interventions using the urine tenofovir test.

Healthcare providers should encourage PrEP use during periods of risk and normalize the choice of PrEP cessation when not needed (i.e. prevention‐effective adherence) [35]. Our study revealed that some TGW resorted to white coat dosing (increasing their adherence to PrEP just before study visits) to conceal non‐adherence [36]. This behaviour may be due to social desirability bias, as some TGW may have postponed their appointments until they had resumed taking PrEP to avoid a negative urine tenofovir test. In light of this issue, providers should adhere to the latest guidance from the World Health Organization (WHO), stating that daily PrEP can be discontinued 7 days after potential exposure to HIV [37]. They should also emphasize the value of supportive sexual healthcare at routine clinic visits regardless of past PrEP usage and normalize PrEP discontinuation to prevent white coat dosing. POC urine tenofovir testing is a non‐invasive and cheaper alternative to blood‐based assays, whose benefits outweigh the potential harms with appropriate counselling. While there are limited published reports of white coat dosing among TGW, a study of 281 men who have sex with men in the United States found that this behaviour was rare (1.1% of visits) but was significantly associated with non‐disclosure of HIV serostatus before sexual intercourse [36]. Our findings highlight the importance of maintaining prevention‐effective drug concentrations to decrease the risk of HIV acquisition.

The WHO recommends implementing differentiated PrEP services to support effective PrEP use [37]. As POC urine tenofovir testing is scaled up to support PrEP adherence, providers should provide clear and comprehensive explanations regarding the distinctions between interpreting POC HIV self‐tests and urine tenofovir tests. Specifically, it should be explained to PrEP users that two lines on an HIV oral‐fluid self‐test device signify the presence of HIV. Conversely, two lines on the current version of the urine test strip (prior to marketing) indicate the absence of tenofovir. This can confuse HIV self‐test users unfamiliar with this novel urine test [18]. Of note, the final marketed urine tenofovir test will likely be backed to more clearly indicate tenofovir detection (e.g. with a plus sign, a smiling fact or a line). Therefore, provider training should be implemented before the rollout of POC urine tenofovir tests to ensure efficient and accurate interpretation by providers and users. Additionally, client‐centred adherence counselling should include culturally sensitive approaches incorporating shared problem‐solving tailored to individual needs. For instance, in Uganda, participants were able to exert autonomy through a focus on individual needs rather than the concept of shared decision‐making, which may be more familiar in a US or European context.

This study offers several strengths, including being the first qualitative evaluation of urine tenofovir testing and drug‐level feedback for promoting oral PrEP adherence among TGW. To our knowledge, it is also the first investigation to provide detailed insights into perceptions and experiences related to POC urine testing for TGW. Our study adds valuable insights to the existing knowledge base for this population. One of the authors identifies as transgender, which aided our interpretation of the data. Additionally, we involved transgender colleagues in various aspects, such as reviewing data collection instruments, recruiting and retaining participants, delivering the intervention, reviewing the manuscript and disseminating the results [16]. However, it is essential to acknowledge the limitations of this work, namely that its findings may not reflect the “real world” implementation of PrEP delivery due to the clinical trial setting. It is important to note that our findings are not intended to be generalizable as they stem from qualitative research. However, they may inform POC urine tenofovir testing for TGW in other settings.

## 5 CONCLUSIONS

Urine tenofovir testing and supportive adherence counselling with drug‐level feedback could motivate PrEP adherence among TGW at risk of HIV acquisition. Clear explanations of test results by study staff will facilitate its utility. Ongoing and future studies should utilize client‐centred counselling and include TGW as equal partners in community‐based participatory research to advance health equity for this key population and facilitate the achievement of HIV epidemic control.

## COMPETING INTERESTS

AM received a research grant from Gilead Sciences, Inc. for work unrelated to the submitted project. JEH serves as a consultant for Merck.

## AUTHORS’ CONTRIBUTIONS

AM, NCW and JEH conceptualized the study. TRM and AM supervised qualitative data collection and study implementation. AM and JM read the interview transcripts. AM wrote the initial draft. JEH, MAW and NCW reviewed drafts and provided substantial edits. MG generously donated urine tenofovir test kits. All authors carefully reviewed and approved the final version of the manuscript.

## FUNDING

This work was supported through research grants from the United States National Institute of Mental Health (R34MH121084 to AM and K24MH114732 to JEH).

## DISCLAIMER

This paper represents the opinions of the authors and does not necessarily represent the official views of the National Institutes of Health.

## Data Availability

The data that support the findings of this study are available from the corresponding author upon reasonable request.
